# Supplementary figures and images for: Modern Acinetobacter baumannii clinical isolates replicate inside spacious vacuoles and egress from macrophages
Source: PLoS Pathog. 2021 Aug 9;17(8):e1009802. doi: 10.1371/journal.ppat.1009802 (PMC8376066; doi:10.1371/journal.ppat.1009802)

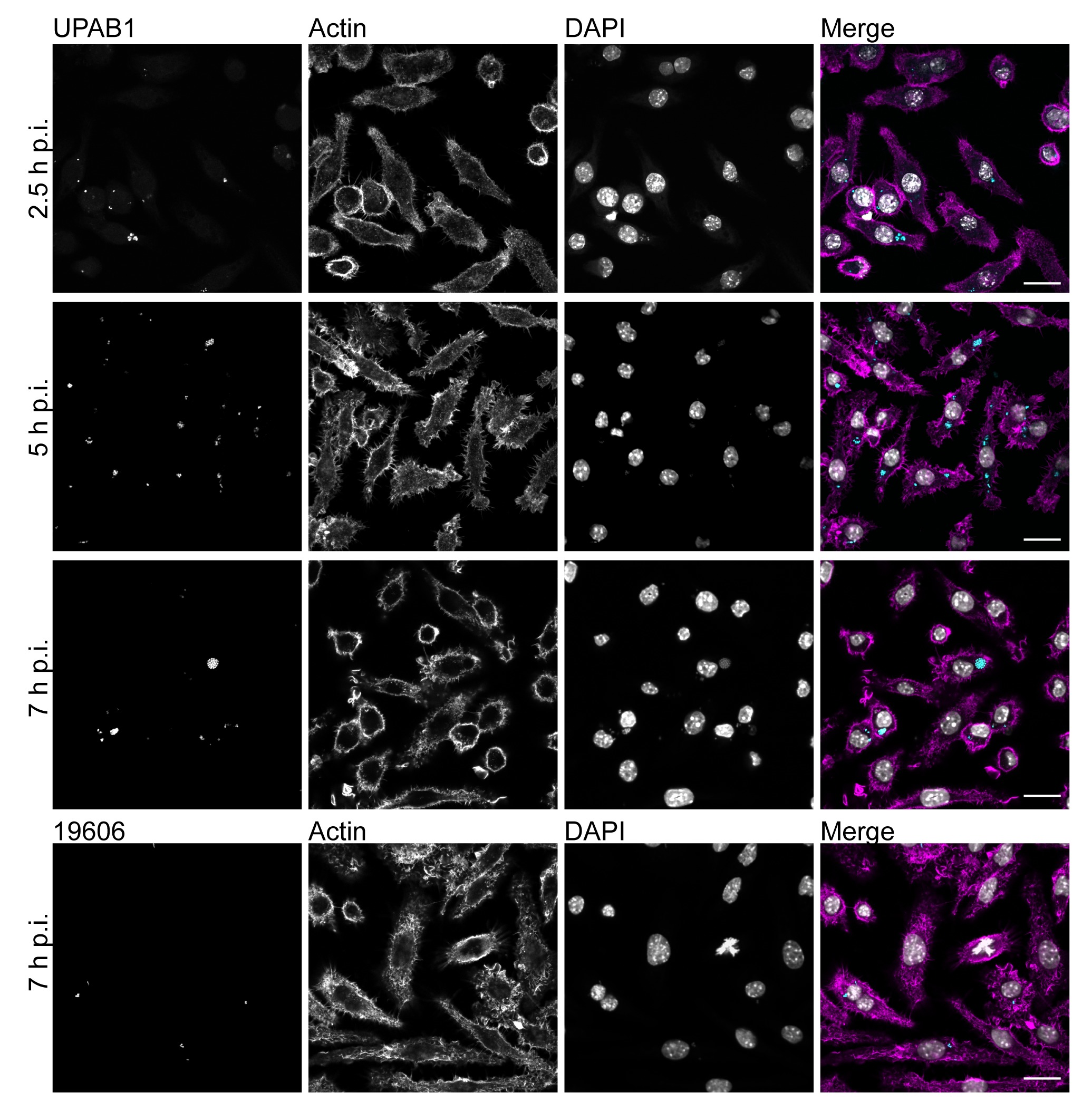

Supplement: S1 Fig — Representative images of infected cells at the indicated time points. Samples were stained for cell nuclei (grey), actin (magenta) and Acinetobacter GFP (cyan). Individual channels (greyscale) and merged images are shown. Bars: 20 μm. (TIF) [file ppat.1009802.s001.tif]

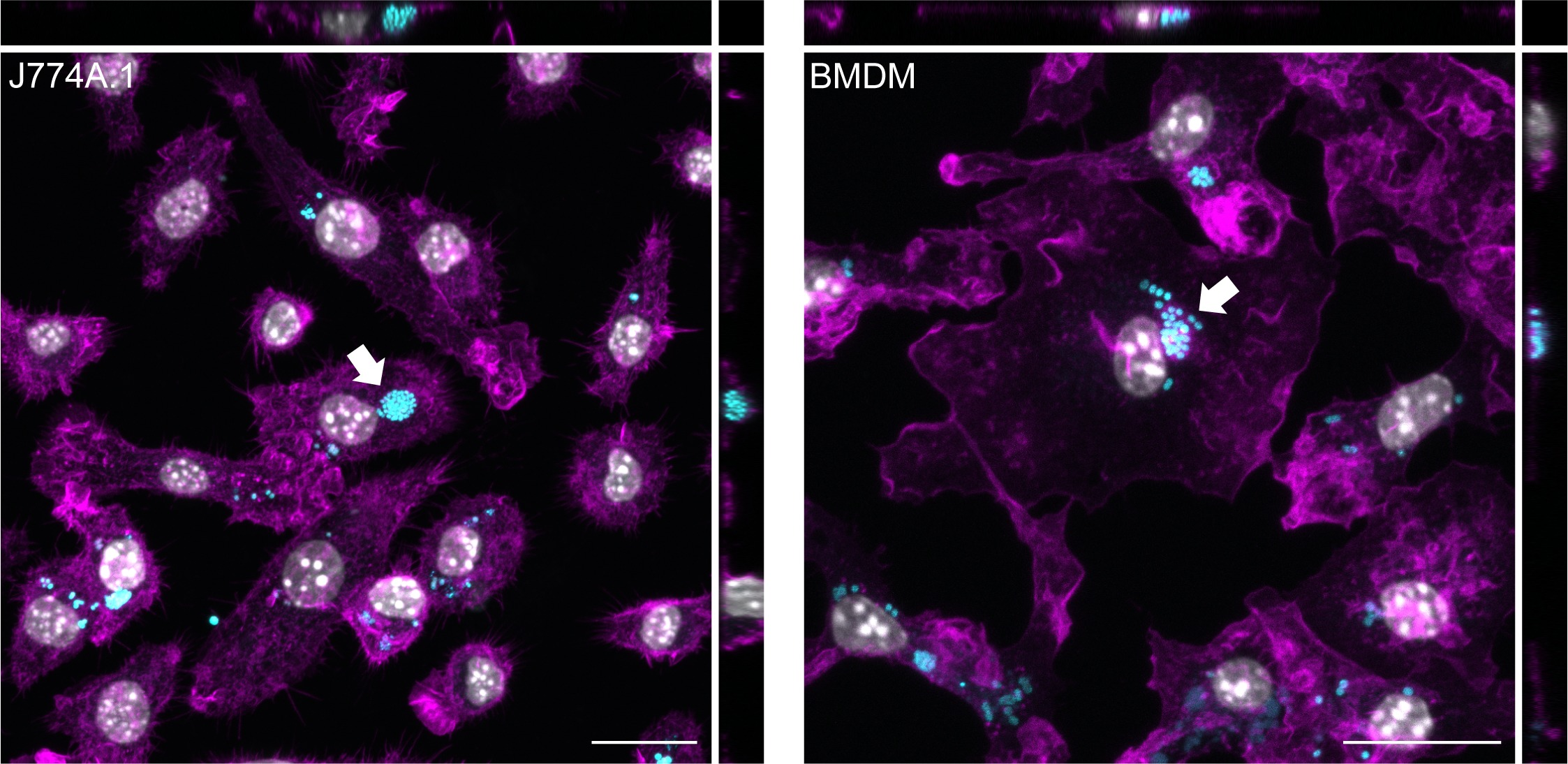

Supplement: S2 Fig — Representative Z-projections with orthogonal views of infected J774A.1 cells (left panel) or BMDM (right panel) at 7 and 5 h p.i., respectively. Samples were stained for cell nuclei (grey), actin (magenta) and Acinetobacter GFP (cyan). Bars: 20 μm. White arrows indicate the vacuole. (TIF) [file ppat.1009802.s002.tif]

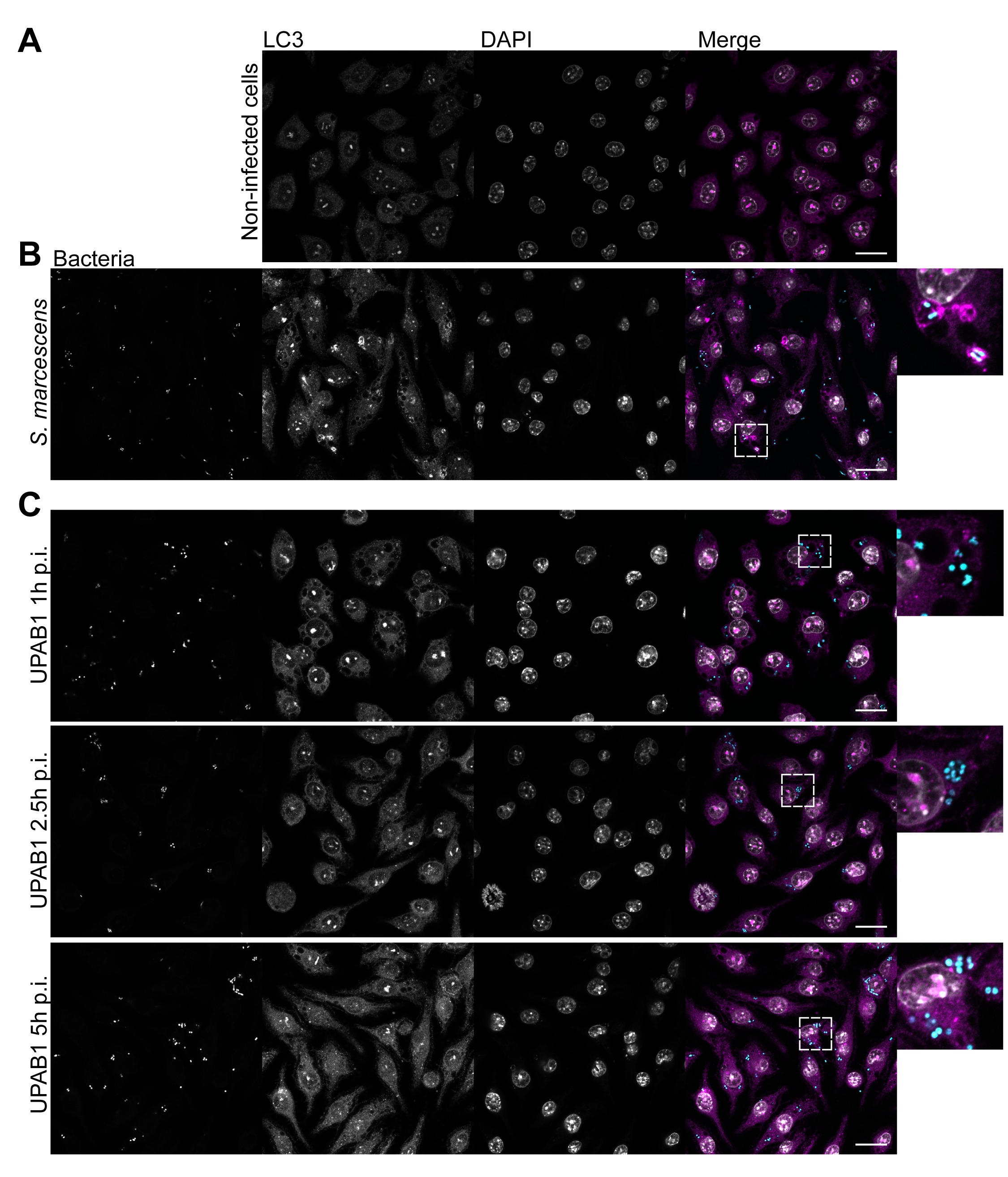

Supplement: S3 Fig — Representative images of J774A.1 macrophages (A) non-infected, (B) Serratia marcescens-infected (2 h p.i.), or (C) UPAB1-infected (1, 2.5 and 5 h p.i.). Samples were stained for cell nuclei (grey), LC3 (magenta) and bacteria expressing GFP (cyan). Individual channels (greyscale) and merged images are shown. Insets show a higher magnification of the area indicated by the white box. Bars: 20 μm. (TIF) [file ppat.1009802.s003.tif]

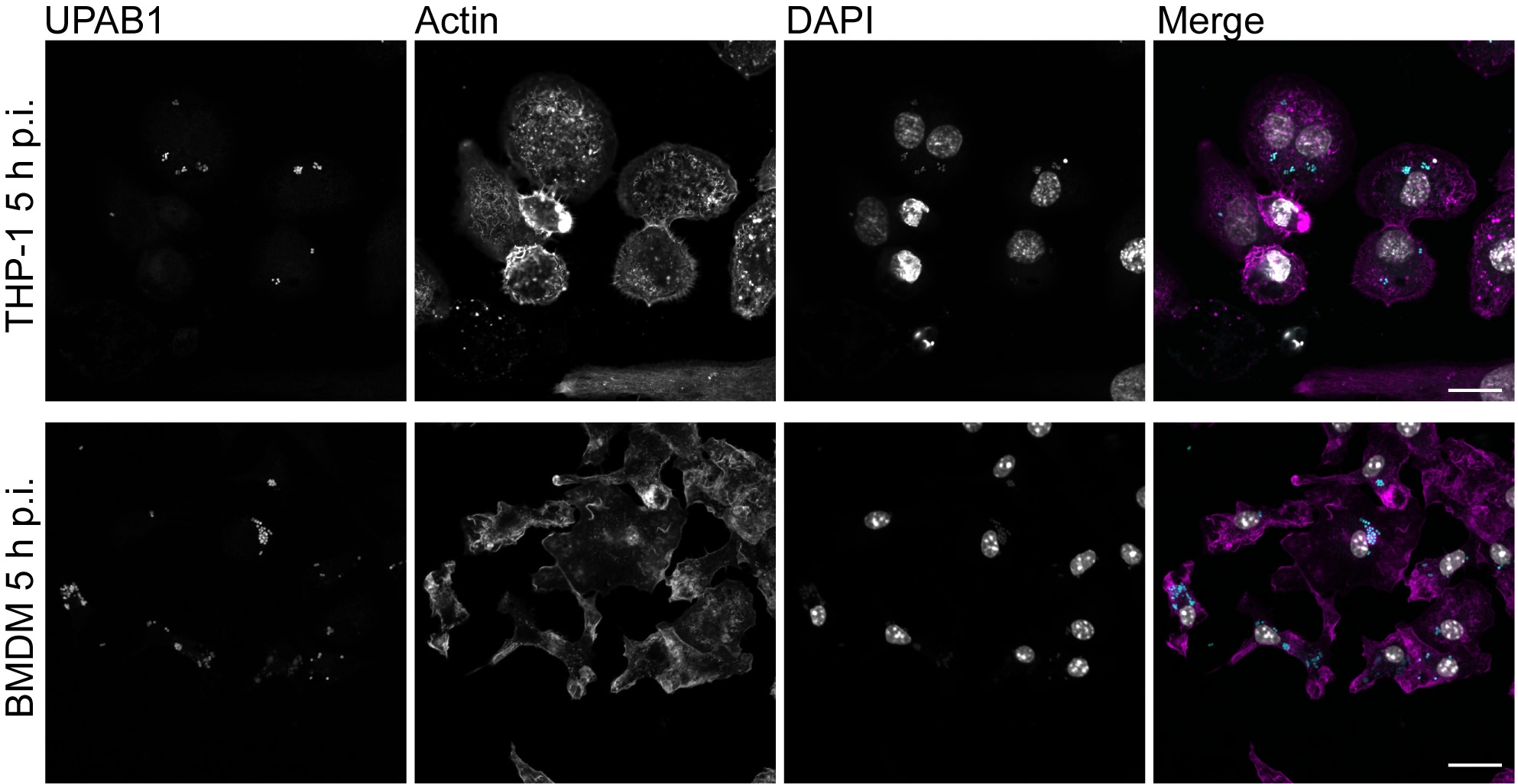

Supplement: S4 Fig — Representative images of infected THP-1 (upper panels) or BMDM (lower panels) cells at 5 h p.i. Samples were stained for cell nuclei (grey), actin (magenta) and UPAB1 GFP (cyan). Individual channels (greyscale) and merged images are shown. Bars: 20 μm. (TIF) [file ppat.1009802.s004.tif]

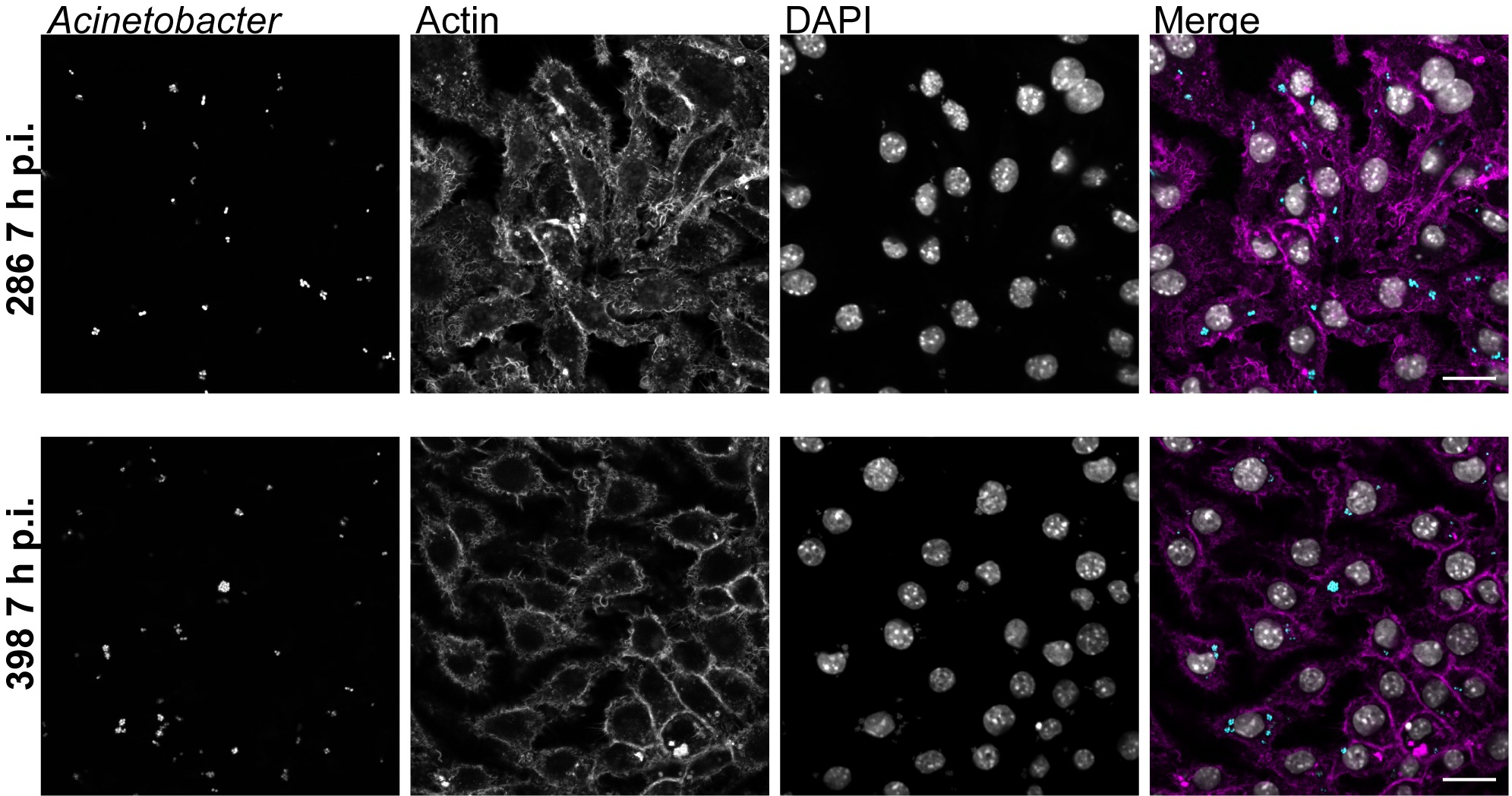

Supplement: S5 Fig — Representative images of infected cells with the indicated strains. Samples were stained for cell nuclei (grey), actin (magenta) and Acinetobacter GFP (cyan). Individual channels (greyscale) and merged images are shown. Bars: 20 μm. (TIF) [file ppat.1009802.s005.tif]
