# Supplementary material for: Modern Acinetobacter baumannii clinical isolates replicate inside spacious vacuoles and egress from macrophages
Source: PLoS Pathog. 2021 Aug 9;17(8):e1009802. doi: 10.1371/journal.ppat.1009802 (PMC8376066; doi:10.1371/journal.ppat.1009802)
Supplement: S1 Table — (DOCX) [file ppat.1009802.s006.docx]

**Table S1. Bacterial strains.**

| **Bacterial strain** | **Description** | **Reference** | |
| --- | --- | --- | --- |
| ***Acinetobacter baumannii*** | | | |
| UPAB1 | MDR urine isolate with pAB5 plasmid | [1] | |
| UPAB1p- | Derivative strain without pAB5 | [1] | |
| 19606 | Urinary tract infection isolate | [2] | |
| AbCAN2 | Bone clinical isolate | [3] | |
| 286 | *A.b.* HUC88.01. Urinary tract infection clinical isolate | This work. | |
| 398 | *A.b.* 1955182 | IHMA Labs. | |
| 413 | Clinical isolate. ACATPM 4061-2 | U. of Pittsburgh. | |
| 414 | Clinical isolate. ACATPM 4079 | U. of Pittsburgh. | |
| UPAB1 GFP | GFP-expressing transconjugant | This work. | |
| 19606 GFP | GFP-expressing transconjugant | This work. | |
| 286 GFP | GFP-expressing transconjugant | This work. | |
| 398 GFP | GFP-expressing transconjugant | This work. | |
| UPAB1pAB3 | Derivative strain without pAB5 and pAB3 conjugated | [1] | |
| UPAB1pAB5*Δ*h-ns | UPAB1 containing an unmarked *h-ns* deletion in pAB5 plasmid | [4] | |
| *ΔhlyB1* | UPAB1 strain with an unmarked deletion of the *hlyB1* (D1G37_13690) gene corresponding to the T1SSa | This work. | |
| *ΔhlyD2* | UPAB1 strain with an unmarked deletion of the *hlyD2* (DG137_00070) gene corresponding to the T1SSb | This work. | |
| Δ*hlyB1,*Δ*hlyD2* | UPAB1 strain with an unmarked deletion of the *hlyB1* (D1G37_13690) and *hlyD2* (DG137_00070) genes | This work. | |
| ***Serratia marcescens*** | | | |
| *Serratia marcescens* GFP | RM 66262 expressing GFP. | | [5] |
| ***Escherichia coli*** | | | |
| Stellar Competent Cells | *F–, endA1, supE44, thi-1, recA1, relA1, gyrA96, phoA, Φ80d lacZΔ M15, Δ(lacZYA-argF) U169, Δ(mrr-hsdRMS-mcrBC), ΔmcrA, λ–* | Clontech. | |

**References**

1. Di Venanzio G, Flores-Mireles AL, Calix JJ, Haurat MF, Scott NE, Palmer LD, et al. Urinary tract colonization is enhanced by a plasmid that regulates uropathogenic Acinetobacter baumannii chromosomal genes. Nat Commun. 2019;10: 1100. doi:10.1038/s41467-019-10706-y

2. Hugh R, Reese R. Designation of the type strain for Bacterium anitratum Schaub and Hauber 1948. Int J Syst Bacteriol. 1967;17: 245–254. doi:10.1099/00207713-17-3-245

3. Lopez J, Ly PM, Feldman MF. The tip of the VgrG spike is essential to functional type VI secretion system assembly in acinetobacter baumannii. MBio. 2020;11: 1–14. doi:10.1128/mBio.02761-19

4. Benomar S, Di Venanzio G, Feldman MF. Plasmid-encoded H-NS controls extracellular matrix composition in a modern Acinetobacter baumannii urinary isolate. bioRxiv. 2021; 2021.05.19.444899. doi:10.1101/2021.05.19.444899

5. Fedrigo G V, Campoy EM, Di Venanzio G, Colombo MI, García Véscovi E. Serratia marcescens Is Able to Survive and Proliferate in Autophagic-Like Vacuoles inside Non-Phagocytic Cells. May RC, editor. PLoS One. 2011;6: 15. doi:10.1371/journal.pone.0024054
