# Supplementary material for: Modern Acinetobacter baumannii clinical isolates replicate inside spacious vacuoles and egress from macrophages
Source: PLoS Pathog. 2021 Aug 9;17(8):e1009802. doi: 10.1371/journal.ppat.1009802 (PMC8376066; doi:10.1371/journal.ppat.1009802)
Supplement: S2 Table — (DOCX) [file ppat.1009802.s007.docx]

**S2 Table. List of primers used in this study.**

| **Primers** | |
| --- | --- |
| UpstB1F | GCGAGTCAAGCAGACCCAATTC |
| UpstB1R | CAGCTCCAGCCTACACAATCGCTGGTTGTACTTCGAACCCCTGAAT |
| DownstB1F | GAACTAAGGAGGATATTCATATGATGAGCGAACAACAACAAGAATTTACC |
| DownstB1R | TCTTTTACGACACCGCGCAC |
| UpstD1F | CTTGCGATCCGCCATTTAAAAATTCG |
| UpstD1R | CAGCTCCAGCCTACACAATCGCTGAATTGCCCCCTTGGCTCAC |
| DownstD1F | GGAATAGGAACTAAGGAGGATATTCATATGGAAAAAAAGCCCCGAAA |
| DownstD1R | CTTCGCGACCACGAGTCAC |
| UpstB2F | TAAATCGGTCGCCTGACGTTTC |
| UpstB2R | CAGCTCCAGCCTACACAATCGCTATGAGTTATAAACAGCTGAATTGGGCT |
| DownstB2F | GAACTAAGGAGGATATTCATATGGGCTCAATTTTGAATCCTTGAATAGAAGTATCA |
| DownstB2R | AGCCGATCCAGTACAAGCTCAG |
| UpstD2F | CGTCAACAAGGTCGGCCTAATT |
| UpstD2R | AGCTCCAGCCTACACAATCGCTTAAATCTTCAAAAAATAAGAATTTTACAGTGGATTTAT |
| DownstD2F | GAACTAAGGAGGATATTCATATGCATTCGGCTCCTATTTTTTGCTTTGG |
| DownstD2R | CAACCTCCTCGTGCACATCTC |
| P1 | AGCGATTGTGTAGGCTGGAGCTG |
| P2 | CATATGAATATCCTCCTTAGTTCCTATTCC |
| GFP Fw | TGAGCTCACTAGTGGATCCCCAATTCACTGTTCCTTGC |
| GFP Rv | GGCATGGATGAACTATACAAATAAAAGCTTGGGCCCGGTACC |
| pUCT-lin-Fw | AAGCTTGGGCCCGGTACC |
| pUCT-lin-Rv | GCATGAGCTCACTAGTGGATCC |
