# Supplementary material for: Modern Acinetobacter baumannii clinical isolates replicate inside spacious vacuoles and egress from macrophages
Source: PLoS Pathog. 2021 Aug 9;17(8):e1009802. doi: 10.1371/journal.ppat.1009802 (PMC8376066; doi:10.1371/journal.ppat.1009802)
Supplement: S3 Table — (DOCX) [file ppat.1009802.s008.docx]

**Table S3. List of plasmids used in this study.**

| **Plasmid** | **Description** |
| --- | --- |
| pBAV1k-t5-gfp | published vector [1], kanamycin resistance marker |
| pUC18T-mini-Tn*7*T-Zeo | published vector [2], zeocin resistance marker |
| pUC18T-mini-Tn*7*T-Zeo-GFP | promotor promlac and *gfp* gene from pBAV1k-t5-gfp on pUC18T-mini-Tn*7*T-Zeo backbone, zeocin resistance marker |

**Reference**

1. Bryksin A V., Matsumura I. Rational Design of a Plasmid Origin That Replicates Efficiently in Both Gram-Positive and Gram-Negative Bacteria. Mokrousov I, editor. PLoS One. 2010;5: e13244. doi:10.1371/journal.pone.0013244

2. Ducas-Mowchun K, De Silva PM, Crisostomo L, Fernando DM, Chao TC, Pelka P, et al. Next generation of Tn7-based single-copy insertion elements for use in multi- and pan-drug-resistant strains of Acinetobacter baumannii. Appl Environ Microbiol. 2019;85. doi:10.1128/AEM.00066-19
