# Supplementary material for: Modern Acinetobacter baumannii clinical isolates replicate inside spacious vacuoles and egress from macrophages
Source: PLoS Pathog. 2021 Aug 9;17(8):e1009802. doi: 10.1371/journal.ppat.1009802 (PMC8376066; doi:10.1371/journal.ppat.1009802)
Supplement: Attachment — Submitted filename: Cover letter.docx [file ppat.1009802.s010.docx]

Washington University in St. Louis School of Medicine

Department of Molecular Microbiology,

660 S. Euclid Avenue, Campus Box 8230,

Saint Louis, Missouri 63110

June 30, 2021

Dear Editor,

Please find enclosed the revised version of our manuscript entitled “Modern *Acinetobacter baumannii* clinical isolates replicate inside spacious vacuoles and egress from macrophages” by Sycz and Di Venanzio *et al*.

We would like to thank the reviewers for their positive and constructive comments. We have addressed their concerns and added new experiments when necessary. Directly below this letter, you will find our detailed responses to the reviewers’ concerns.

We hope you will find this revised version suitable for publication in PLoS Pathogens.

Sincerely,

Mario F. Feldman, Ph.D.

Professor of Molecular Microbiology

Washington University School of Medicine

Department of Molecular Microbiology

[mfeldman@wustl.edu](mailto:mfeldman@wustl.edu)

Tel. 314-747-4473

Reviewer's Responses to Questions

**Part I - Summary**
Please use this section to discuss strengths/weaknesses of study, novelty/significance, general execution and scholarship.

Reviewer #1: In this manuscript, Sycz and colleagues describe a phenomenon in which several Acinetobacter baumannii clinical isolates (such as UPAB1), unlike common laboratory stains, replicate in the vacuoles of macrophages and eventually lead to lytic cell death. The intracellular survival of UPAB1 relies on a large conjugative plasmid (pAB5) and its type I secretion system (T1SS) regulated by pAB5. The study is quite straightforward and the conclusion is justified. The importance of this study is that it urges the field to focus on the clinical stain as it clearly differs from the long and commonly used laboratory strain in the virulence and intracellular trafficking properties. The observations reported in this study will serve as a good foundation for the field to further analyze the pathogenesis mechanism of Acinetobacter baumannii, which is more relevant to understand the real-world infections caused by this important pathogen. However, the study is still of descriptive nature and lacks in-depth insights into the mechanism underlying the unique intracellular life cycle of the new Acinetobacter baumannii strain.

Reviewer #2: In this manuscript, Feldmann and colleagues demonstrate that Acinetobacter baumannii can survive intracellularly in macrophages. Previous studies with A. baumannii had largely focused on a 19606, a urinary isolate obtained in 1967. This strain does not survive in macrophages, which led to the idea that A. baumannii is an "extracellular opportunistic pathogen". The findings reported here turn this idea upside down! The manuscript is very well-written, the experiments easy to follow and the conclusions are justified. Only minor weaknesses were noted, specifically in figure presentation and lack of clarity about the prevalence of the intracellular replication phenotype amongst A. baumannii isolates.

**Part II – Major Issues: Key Experiments Required for Acceptance**
Please use this section to detail the key new experiments or modifications of existing experiments that should be absolutely required to validate study conclusions.

Generally, there should be no more than 3 such required experiments or major modifications for a "Major Revision" recommendation. If more than 3 experiments are necessary to validate the study conclusions, then you are encouraged to recommend "Reject".

Reviewer #1: 1. I am not convinced that half of the vacuoles surrounding Acinetobacter baumannii UPAB1 have double-membrane structures, according to the right panel of Fig 1B. The lipid bilayer shown in this panel does not seem to tell the difference between the left and the right panel. More convincing data, including appropriate controls and statistics are needed to conclude this point. Also, the logic flow is a little strange. Examination of the LC3 marker on the ACVs should follow immediately after the discovery of the double-membrane structure, rather than follows the description of bacterial CFU in different macrophages. The authors should address what is the nature of the double-membrane structure as it is counterintuitively LC3-negative.

We agree with the reviewer that the logic flow in the paper was not optimal. We modified the manuscript as suggested.

A double-membrane structure is visible is some vacuoles. Unfortunately, we cannot properly quantify the percentage of vacuoles displaying double membranes from the TEM images. Therefore, we changed “about half” to “some” in the text. Despite the presence of double membranes, we found that the ACVs are LC3 negative. This is puzzling. It has been reported that double membrane structures can be formed in an ATG3-independent manner without LC3 recruitment in fibroblasts [1]. Moreover, several intracellular pathogens manipulate the host vesicular pathways, particularly autophagy, in different ways to create a replicative niche and/or avoid the host elimination [2]. We now discuss these findings in the text (lines 275-284)

The Salcedo group has reported that the ACV in epithelial cells are LC3 negative, but in their model ACV are surrounded by single membranes. Understanding the fascinating vacuole created by A. baumannii in different cell types is the subject of on-going experiments in the lab, and we believe that the elucidation of the nature of the ACVs is out of the scope of the present work.

2. Which kind of lytic cell death is induced by UPAB? Markers of different cell-death types should be checked. Further, what about other clinical strains and the UPAB strain lacking pAB5 or T1SS, and do they induce similar lytic cell death? It is reasonable to speculate that the decrease in CFU of 19606 or pAB5-deficient UPAB may be due to the faster lytic death of macrophages. Therefore, more details and mechanisms should be provided.

Although the decrease of intracellular CFUs of 19606 could be explained by a faster lytic death, previous literature has shown that A. baumannii 19606 is degraded by host cells [3–5]. We are working on the extensive characterization of the egress mechanism of A. baumannii. We hope to be able to publish these results in the future.

3. Give more information about the LCP plasmid. In addition to transcriptional regulation, are there other genes involved in bacterial virulence? What are the key transcription factors? Could the macrophage replicating phenotype be reconstituted in 19606 by putting back the pAB5 plasmid?

We thank the reviewer for their comment. We have unsuccessfully tried to introduce pAB5 into 19606 employing different methods. We did successfully introduce another plasmid, pAB3, into UPAB1, and this plasmid did not support replication of UPAB1 inside macrophages. pAB3 and pAB5 belong to the same family of plasmids, although they differ in the content of transcriptional factors, antibiotic resistance, and other additional sequences.

The pAB5 plasmid was first characterized in 2019 [6]. Based on our transcriptomic and proteomic data, our favorite hypothesis is that pAB5 enhances intracellular replication of UPAB1 by regulating the expression of chromosomally encoded genes. A recent pre-print from our group, available in BioRxiv [7], describes the key transcriptional factors present in pAB5 and shows that pAB5-encoded H-NS transcriptional regulator represses the synthesis of the exopolysaccharide PNAG and the expression of a previously uncharacterized three-gene cluster that encodes a protein belonging to the CsgG/HfaB family. We therefore tested if this regulator will also impact the ability of UPAB1 to replicate inside macrophages.

We found that pAB5 lacking H-NS does not longer support UPAB1 intracellular replication, supporting our hypothesis. These new results exploring the role of LCPs and HNS were incorporated in Figure 6A and throughout the text.

4. A key aspect of this study is to explore the mechanism of UPAB replication in macrophages. However, data addressing this question are weak and not sufficient. More insights into the mechanism should be provided.

Although we agree with the reviewer that the mechanistic insights of Acinetobacter replication in macrophages are extremely important, we have provided the first evidence of Acinetobacter baumannii replication and exit inside macrophages. In this first publication, we show that several modern A. baumannii clinical isolates, including the recent clinical urinary isolate UPAB1, persist and replicate inside macrophages within spacious vacuoles. We show that some of those vacuoles have a double membrane although no LC3 co-localization was found at any time p.i. We believe that we provide significant insights into the mechanisms from “the bacterial side”. We show that intracellular replication of UPAB1 is dependent on a functional type I secretion system (T1SS) and pAB5, a large conjugative plasmid that controls the expression of several chromosomally-encoded genes. We also show that pAB3, the founding member of the LCP family, does not support UPAB1 replication, and that the HNS transcriptional factor encoded by pAB5 is required for UPAB1 survival inside macrophages. Finally, we show that UPAB1 escapes from the infected macrophages by a lytic process. We believe this data will greatly impact the Acinetobacter field and we hope that, in the future, we and others will provide additional mechanistic details.

5. How about the life cycle of the different A. baumannii strains in epithelial cells? Is that similar to macrophages?

We thank the reviewer for the question. The Salcedo lab has recently published a pre-print describing Acinetobacter replication in epithelial cells [8]. We have discussed their results in the text.

Reviewer #2: (No Response)

**Part III – Minor Issues: Editorial and Data Presentation Modifications**
Please use this section for editorial suggestions as well as relatively minor modifications of existing data that would enhance clarity.

Reviewer #1: (No Response)

Reviewer #2: (1) In Figure 2 and Figure 4, the authors show that UPAB1, 286, 398, 413 and 414 isolates are all capable of limited replication in J774A.1 macrophages. Therefore all recent clinical isolates tested here are capable of replicating inside macrophages. How representative is this phenomenon? Are there recent clinical isolates that were not able to survive and replicate for example?

We thank the reviewer for the comment. We have added replication data from strain AbCAN2 [9]. This strain is a recent isolate from a bone infection and was not able to replicate in J774 macrophages. Therefore, not all the new clinical isolates replicated in macrophages. We have modified Figure 4 and the text accordingly.

(2) The confocal images do not do the findings any justice. They are too small and it is difficult to see any detail. PLOS Pathogens does not have a page limit - please make them larger. Also individual channels should be shown in greyscale, as well as an overlay of the individual channels. See a number of articles on how to present fluorescence microscopy images for color blind readers and to improve clarity.
https://journals.plos.org/plosbiology/article?id=10.1371/journal.pbio.3001161
<https://www.molbiolcell.org/doi/10.1091/mbc.e11-09-0824>

We thank the reviewer for this comment. All microscopy figures have been changed according to the reviewer guidelines.

(3) Lines 48-51. Reference required.

The reference was added.

(4) Line 121. Pertaining to Figure S2C. Two representative images are shown from 5 h p.i. and none from 1 h p.i. Please include an image of the early timepoint for comparison.

We thank the reviewer for this comment. We have modified Figure S2 and added earlier time points for comparison.

(5) Figure 5C. Why was LDH release not measured until 24 h p.i.? Bacterial release occurs at 5-8 h p.i., which should be coincident with LDH release. Please include LDH release data for earlier timepoints, or explain in the text why it was not done at earlier timepoints.

We thank the reviewer for this comment. No LDH activity was detected at 8 or 12 h p.i. The lysis only occurs in a proportion of the cells, and therefore we believe this is a problem of detection for the method. We clarified in the text (lines 185-187)

(6) Line 204. The Salcedo lab has recently reported similar findings in epithelial cells on BioRXiv. Please cite this article rather than "personal communication".

The line was modified to reference the BioRXiv article.

References

1.	Tsuboyama K, Koyama-Honda I, Sakamaki Y, Koike M, Morishita H, Mizushima N. The ATG conjugation systems are important for degradation of the inner autophagosomal membrane. Science (80- ). 2016;354: 1036–1041. doi:10.1126/science.aaf6136

2.	Kimmey JM, Stallings CL. Bacterial Pathogens versus Autophagy: Implications for Therapeutic Interventions. Trends in Molecular Medicine. Elsevier Ltd; 2016. pp. 1060–1076. doi:10.1016/j.molmed.2016.10.008

3.	Wang Y, Zhang K, Shi X, Wang C, Wang F, Fan J, et al. Critical role of bacterial isochorismatase in the autophagic process induced by Acinetobacter baumannii in mammalian cells. FASEB J. 2016; 1–15. doi:10.1096/fj.201500019R

4.	Konstantinidis T, Kambas K, Mitsios A, Panopoulou M, Tsironidou V, Dellaporta E, et al. Immunomodulatory role of clarithromycin in Acinetobacter baumannii infection via formation of neutrophil extracellular traps. Antimicrob Agents Chemother. 2016;60: 1040–1048. doi:10.1128/AAC.02063-15

5.	Bist P, Dikshit N, Koh TH, Mortellaro A, Tan TT, Sukumaran B. The Nod1, Nod2, and Rip2 axis contributes to host immune defense against intracellular Acinetobacter baumannii infection. Infect Immun. 2014;82: 1112–1122. doi:10.1128/IAI.01459-13

6.	Di Venanzio G, Flores-Mireles AL, Calix JJ, Haurat MF, Scott NE, Palmer LD, et al. Urinary tract colonization is enhanced by a plasmid that regulates uropathogenic Acinetobacter baumannii chromosomal genes. Nat Commun. 2019;10: 1–13. doi:10.1038/s41467-019-10706-y

7.	Benomar S, Di Venanzio G, Feldman MF. Plasmid-encoded H-NS controls extracellular matrix composition in a modern 1 Acinetobacter baumannii urinary isolate 2 3. bioRxiv. 2021; 2021.05.19.444899. doi:10.1101/2021.05.19.444899

8.	Rubio T, Gagné S, Debruyne C, Dias C, Cluzel C, Mongellaz D, et al. Incidence of an intracellular multiplication niche amongst Acinetobacter baumannii clinical isolates. bioRxiv. 2021; 2021.04.15.439986. doi:10.1101/2021.04.15.439986

9.	Lopez J, Ly PM, Feldman MF. The tip of the VgrG spike is essential to functional type VI secretion system assembly in acinetobacter baumannii. MBio. 2020;11: 1–14. doi:10.1128/mBio.02761-19

PLOS authors have the option to publish the peer review history of their article ([what does this mean?](https://journals.plos.org/plospathogens/s/editorial-and-peer-review-process#loc-peer-review-history)). If published, this will include your full peer review and any attached files.


If you choose “no”, your identity will remain anonymous but your review may still be made public.


**Do you want your identity to be public for this peer review?** For information about this choice, including consent withdrawal, please see our [Privacy Policy](https://www.plos.org/privacy-policy).

Reviewer #1: No

Reviewer #2: No

Figure Files:

While revising your submission, please upload your figure files to the Preflight Analysis and Conversion Engine (PACE) digital diagnostic tool, [https://pacev2.apexcovantage.com](https://pacev2.apexcovantage.com/). PACE helps ensure that figures meet PLOS requirements. To use PACE, you must first register as a user. Then, login and navigate to the UPLOAD tab, where you will find detailed instructions on how to use the tool. If you encounter any issues or have any questions when using PACE, please email us at [figures@plos.org](mailto:figures@plos.org).

Data Requirements:

Please note that, as a condition of publication, PLOS' data policy requires that you make available all data used to draw the conclusions outlined in your manuscript. Data must be deposited in an appropriate repository, included within the body of the manuscript, or uploaded as supporting information. This includes all numerical values that were used to generate graphs, histograms etc.. For an example see here on PLOS Biology: http://www.plosbiology.org/article/info%3Adoi%2F10.1371%2Fjournal.pbio.1001908#s5.

Reproducibility:

To enhance the reproducibility of your results, we recommend that you deposit your laboratory protocols in protocols.io, where a protocol can be assigned its own identifier (DOI) such that it can be cited independently in the future. Additionally, PLOS ONE offers an option to publish peer-reviewed clinical study protocols. Read more information on sharing protocols at <https://plos.org/protocols?utm_medium=editorial-email&utm_source=authorletters&utm_campaign=protocols>
